# Salvianolic acid B inhibits RAW264.7 cell polarization towards the M1 phenotype by inhibiting NF-κB and Akt/mTOR pathway activation

**DOI:** 10.1038/s41598-022-18246-0

**Published:** 2022-08-16

**Authors:** Tao Zou, Shan Gao, Zhaolan Yu, Fuyong Zhang, Lan Yao, Mengyao Xu, Junxin Li, Zhigui Wu, Yilan Huang, Shurong Wang

**Affiliations:** 1 Department of Pharmacy, Affiliated Hospital of Southwest Medical University, Luzhou, 646000 China; 2 Department of Pharmacology, School of Pharmacy, Southwest Medical University, Luzhou, 646000 China; 3 Department of Pharmacy, Chengdu Second People’s Hospital, Chengdu, 610000 China; 4 Department of Nephrology, The Affiliated Hospital of Southwest Medical University, Luzhou, 646000 China; 5 Department of Pharmacy, People’s Hospital of Deyang City, Deyang, 618000 China

**Keywords:** Biochemistry, Cell biology, Molecular biology

## Abstract

M1 macrophages secrete a large number of proinflammatory factors and promote the expansion of atherosclerotic plaques and processes. Salvianolic acid B (Sal B) exerts anti-inflammatory, antitumor and other effects, but no study has addressed whether Sal B can regulate the polarization of macrophages to exert these anti-atherosclerotic effects. Therefore, we investigated the inhibition of Sal B in M1 macrophage polarization and the underlying mechanism. The effects of different treatments on cell viability, gene expression and secretion of related proteins, phenotypic markers and cytokines were detected by MTT and western blot assays, RT‒qPCR and ELISAs. Cell viability was not significantly changed when the concentration of Sal B was less than 200 μM, and Lipopolysaccharide (LPS) (100 ng/mL) + interferon-γ (IFN-γ) (2.5 ng/mL) successfully induced M1 polarization. RT‒qPCR and ELISAs indicated that Sal B can downregulate M1 marker (Inducible Nitric Oxide Synthase (iNOS), Tumor Necrosis Factor-α (TNF-α), and Interleukin-6 (IL-6)) and upregulate M2 marker (Arginase-1 (Arg-1) and Interleukin-10 (IL-10)) expression. Western blotting was performed to measure the expression of Nuclear Factor-κB (NF-κB), p-Akt, p-mTOR, LC3-II, Beclin-1, and p62, and the results suggested that Sal B inhibits the M1 polarization of RAW264.7 macrophages by promoting autophagy via the NF-κB signalling pathway. The study indicated that Sal B inhibits M1 macrophage polarization by inhibiting NF-κB signalling pathway activation and downregulating Akt/mTOR activation to promote autophagy.

## Introduction

Atherosclerosis (AS) is an important cause of cardiovascular disease^1^. The pathogenesis and aetiology of AS has been explained by the inflammatory theory proposed by Ross^2^, which suggests that “AS is an inflammatory disease”. The inflammatory response is involved throughout the occurrence and development of AS, which involves various inflammatory factors and cells^3^, and the inflammatory process is involved in the rupture of plaques and the formation of thrombi^4^. Macrophages play critical roles in the development and regression of AS^5^ and induce M1 (classically activated macrophages) and M2 (alternatively activated macrophages) macrophage subtype acquisition in response to different stimuli^6^. M1 macrophages can promote the expansion and progression of atherosclerotic plaques^7,8^. M2 macrophages clear ox-LDL and promote cholesterol excretion, enhancing the inflammatory environment of atherosclerotic plaques^9^, and exert antiatherogenic effects through inflammatory regression, exocytosis, and tissue repair^10,11^. The aforementioned studies suggested that regulation of the polarization of macrophages in atherosclerotic lesions may be an effective method for preventing and treating AS.

Autophagy is a protective mechanism in a body under stress and is realized mainly through the phagocytosis and degradation of damaged organelles and excess proteins in the body, which can help the cell survive stress caused by an adverse environment^12^. Some studies have found that autophagy plays a vital role in the polarization of macrophages and that autophagy in macrophages exerts anti-atherosclerotic effects by inhibiting inflammation^13^. Moreover, a study indicated that β-glucan promoted macrophage M1 polarization by inhibiting autophagy^14^. These studies suggested that autophagy may play an important role in the process by which Sal B inhibits macrophage M1 polarization. Nuclear factor (NF)-κB is a protein product that exerts an important effect on the inflammatory process in the body. Several studies^15–17^ showed that NF-κB is involved in atherosclerosis by regulating inflammation in the body, and one of the classical activation pathways of macrophages is NF-κB^18^.

Salvianolic acid B (Sal B), whose molecular formula is C_36_H_30_O_16_, is extracted from the dried roots and rhizomes of Danshen in the labiate family. Sal B is the main water-soluble and most abundant compound in these plants^19^. Several studies confirmed that Sal B exerts anti-inflammatory, antifibrotic, and antitumor effects^20–22^. In terms of cardiovascular function, Sal B attenuated myocardial ischaemia–reperfusion injury and antiatherosclerosis^23–25^; however, whether Sal B regulates the ratio of M1/M2 macrophages was unclear and not studied. Moreover, whether the anti-atherosclerotic mechanisms of Sal B action involve autophagy remains to be fully elucidated. Autophagy and NF-κB are a crucial mechanism and pathway, respectively, involved in regulating atherosclerosis, but the relationship between autophagy and NF-κB remains to be confirmed when Sal B is used to treat atherosclerosis. Therefore, in the present study, we aimed to explore the effect and mechanism of Sal B action on macrophage phenotype acquisition. M1 macrophages were established by LPS and IFN-γ treatment, and changes in autophagy were observed. Then, Sal B was used to pretreat macrophages, and changes in the macrophage phenotype were observed to explore the possible molecular mechanism.

## Materials and methods

### Chemicals and reagents

**Figure 1:**
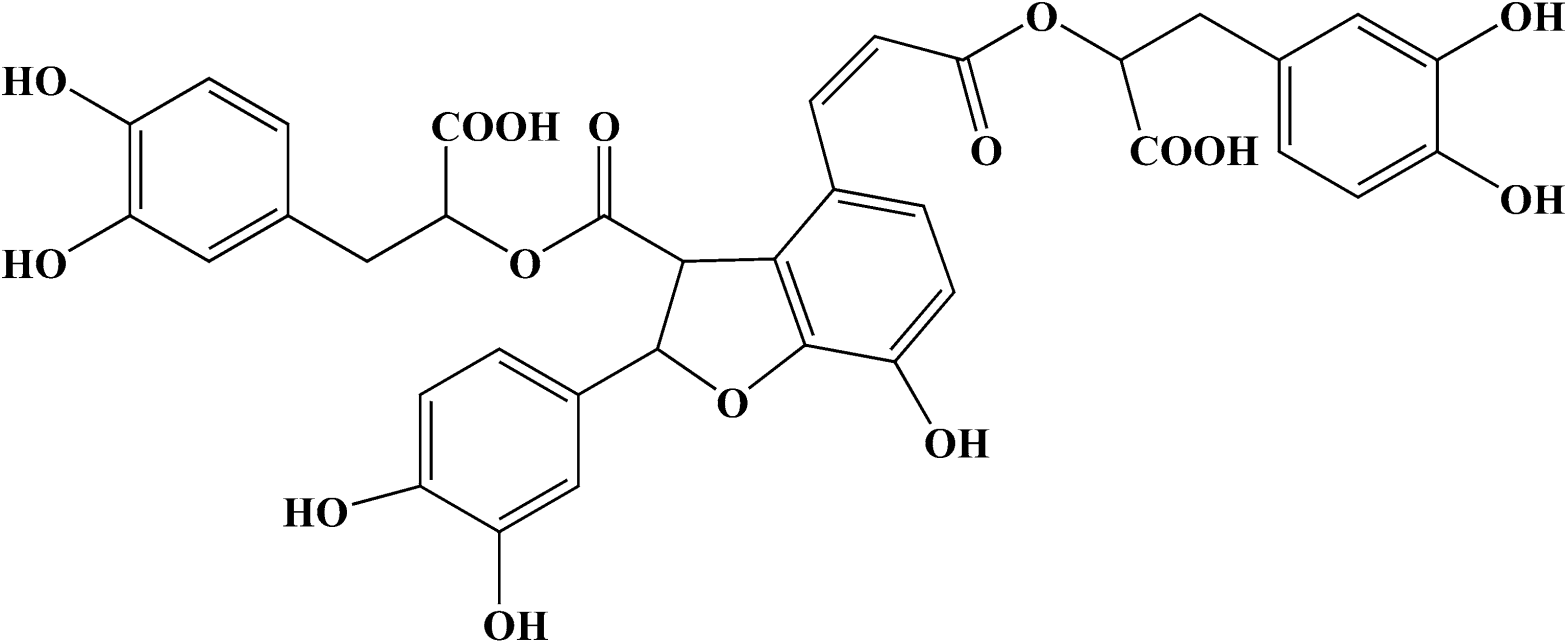
Chemical structure of salvianolic acid B.

Sal B (HPLC ≥ 98%; Fig. 1) was obtained from Chengdu Manster Biotechnology Co., Ltd. (Chengdu, China). RAW264.7 cells were provided by the Cell Bank of the Chinese Academy of Sciences (Manassas, VA, USA). Dulbecco's modified Eagle's medium (DMEM) was purchased from Gibco/Invitrogen. Foetal bovine serum (FBS) was obtained from Thermo Fisher Scientific, Inc. (Waltham, MA, USA). An MTT assay kit, bicinchoninic acid (BCA) protein assay kit, LPS, and insulin were all purchased from the Beyotime Institute of Biotechnology. IFN-γ was obtained from Sino Biological Inc. (Beijing, China). 3-Methyladenine (3-MA) (Selleck, Houston, TX, USA) was used to inhibit autophagy. ELISA kits for mouse interleukin (IL)-6, IL-10, and tumour necrosis factor-α (TNF-α) measurements were obtained from Jiangsu Jingmei Biotechnology Co., Ltd. (Jiangsu, China). A total RNA extraction kit (TRIzol reagent), SYBR Green Real-Time PCR kit were purchased from Foregene Biotechnology (Chengdu, China). iNOS primers, Arg-1, and GAPDH were obtained from Sangon Biotech Co., Ltd. (Shanghai, China). Rabbit anti-LC3-II (cat. no. ab192890; 1:2,000), anti-p62 (cat. no. ab109012; 1:10,000), anti-Akt (cat. no. ab179463; 1:10,000), anti-phosphorylated (p)-Akt (cat. no. ab81283; 1:5000), anti-mammalian target of rapamycin (mTOR; cat. no. ab32028; 1:1000) and anti-p-mTOR (cat. no. ab109268; 1:1000), anti-NF-κB p65 (ab207297) and anti-histone H3 (ab1791) were provided by Abcam (Cambridge, MA, USA). An antibody against Beclin-1 (cat. no. 3495T; 1:1000) was obtained from Cell Signaling Technology, Inc. (Danvers, MA, USA). β-Actin (cat. no. bs-0061R; 1:5000) was purchased from Bioss Biotechnology (Beijing, China), and goat anti-mouse IgG (cat. no. bs-0295GS; 1:2000) was purchased from Beyotime Institute of Biotechnology (Shanghai, China).

### Experimental design

Sal B was dissolved in DMSO at 1 mM, stored in aliquots at − 20 °C, and further diluted (as appropriate) in culture medium. The following cell groups were generated:

1. Control group: RAW264.7 cells were treated with cell culture medium of the same volume and at the same times as in the other groups.
2. LPS + IFN-γ group (M1 group): LPS (100 ng/mL) + IFN-γ (2.5 ng/mL) was added to cultures and incubated for 24 h.
3. Sal B + LPS + IFN-γ group (Sal B group): Sal B at 100, 150, or 200 μM was added and incubated for 1 h followed by LPS (100 ng/mL) + IFN-γ (2.5 ng/mL) treatment for 24 h.
4. Sal B + LPS + IFN-γ + 3-MA group (3-MA group): Sal B at 100, 150, or 200 μM for 1 h followed by LPS (100 ng/mL) + IFN-γ (2.5 ng/mL) + 3-MA (5 mM) treatment for 24 h.
5. Sal B + LPS + IFN-γ + Phorbol Myristate Acetate (PMA) group (PMA group): Sal B at 100, 150, or 200 μM for 1 h followed by LPS (100 ng/mL) + IFN-γ (2.5 ng/mL) + PMA (100 nm/mL) treatment for 24 h.
6. Sal B + LPS + IFN-γ + insulin group (insulin group): Sal B (100, 150, or 200 μM) was added and incubated for 1 h followed by LPS (100 ng/mL) + IFN-γ (2.5 ng/mL) + insulin (1 µg/mL) treatment for 24 h.

### Cell culture

The RAW264.7 cells were cultured in DMEM containing 10% FBS in an incubator at 37 °C with 5% CO_2_.

### Cell viability assay

RAW264.7 cells were cultivated at a density of 5,000 cells per well in 96-well plates for 12 h at 37 °C and then treated with different concentrations of Sal B for 24 h. Then, 5 μL of MTT solution (5 mg/mL) was added to each well and incubated at 37 °C for 3–4 h. The liquid was removed, and DMSO was added to each well. The absorbance was detected with a microplate reader (Bio-Rad 550; Bio-Rad Laboratories, Inc.) at 490 nm. The optical density (OD) was used to determine the percentage of viable cells via the following formula: cell viability (%) = (OD _treatment group_–OD_blank group_/OD_control group_–OD_blank group_) × 100%.

### Enzyme-linked immunosorbent assay

RAW264.7 cells were seeded into a 6-well plate at a density of 20 × 10^4^ cells per well, and then drugs were added as indicated at 37 °C and incubated for 24 h. The culture media were collected, and the cytokine concentrations, which included TNF-α, IL-6, and IL-10, were measured with the corresponding ELISA kit.

### RT‒qPCR

The cells were cultivated in a 6-well plate at a density of 40 × 10^4^ cells per well and incubated at 37 °C for 12 h. Following different treatments, different drugs were added to each well and incubated at 37 °C for 24 h. Total RNA was extracted from the cells in the 6-well plate with TRIzol reagent and was subjected to reverse transcription-quantitative polymerase chain reaction (RT‒qPCR) to measure iNOS and Arg-1mRNA expression. The primer sequences are shown in Table 1. qPCR was performed under the following conditions: 3 min at 95 °C for 1 cycle, 10 s at 95 °C, 30 s at 65 °C for 39 cycles, and 95 °C for 5 s. Changes in the expression of target genes were calculated though the 2^-ΔΔCq^ method^26,27^.

**Table 1:** The primer sequences.

	Forward	Reverse
iNOS	5′-GTTACCATGAGGCTGAAATCC-3′	5′-CCTCTTGTCTTTGACCCAGTAC-3′
Arg-1	5′-CATATCTGCCAAAGACATCGTG-3′	5′-GACATCAAAGCTCAGGTGAATC-3′
GAPDH	5′-GGACCTCATGGCCTACATGG-3′	5′-TAGGGCCTCTCTTGCTCAGT-3′

### Western blot analysis

Western blotting was performed to measure the protein expression of p62, LC3-II, Beclin-1, NF-κB p65, Akt, p-Akt, mTOR, and p-mTOR. RAW264.7 cells were seeded into a six-well cell culture plate at a density of 40 × 10^4^ cells/well. After treatment, the cells were lysed with RIPA lysis buffer (Beyotime Institute of Biotechnology) which contained protease inhibitors, phosphatase inhibitors, and PMSF at 4 °C for 30 min, and then, all soluble lysates were collected by centrifugation at 1.2 × 10^4^ rpm for 30 min at 4 °C. The lysates were boiled at 100 °C for 15 min to denature the proteins, and a BCA kit was used to measure the protein concentration. Protein samples (10 μL per lane) were separated on the basis of molecular mass by SDS–PAGE and then transferred onto a polyvinylidene difluoride membrane. The membranes were blocked with 5% skim milk powder for 1 h at room temperature and then incubated with primary antibodies for 24 h at 4 °C. Subsequently, the membranes were washed three times with Tris-buffered saline with 0.1% Tween-20, followed by incubation with a secondary antibody for 1 h at room temperature. After the membrane was washed three times, an enhanced chemiluminescence reagent (Merck KGaA) was added to the membrane, and the proteins which quantified with a gel imaging system (Bio-Rad Laboratories, Inc.).

### Statistical analyses

ImageJ software, SPSS statistical software (version 17.0; SPSS, Inc., Chicago, IL, USA.), and GraphPad Prism 9.0 software were used for statistical analyses, and all data are presented as the mean ± standard error of the mean. Student's t test was used to compare differences between two groups, and differences between multiple groups were compared by one-way ANOVA. P < 0.05 was indicative of a statistically significant difference.

### Consent for publication

All coauthors of in this research approved the publication of this article.

## Results

### Effect of Sal B on cell viability

**Figure 2:**
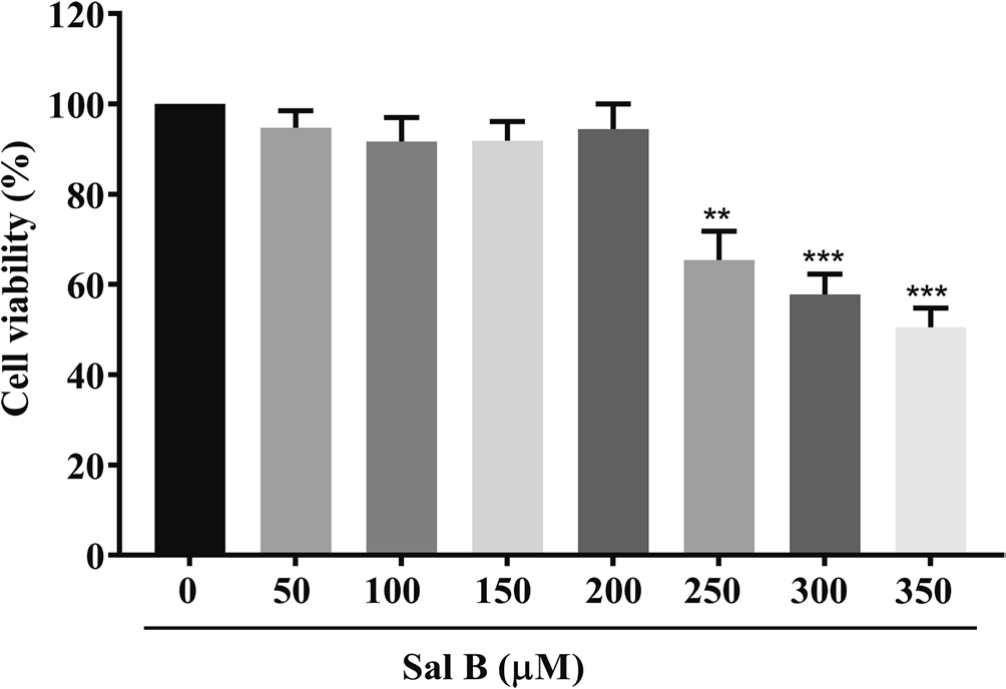
Effects of Sal B on the viability of RAW264.7 cells. An MTT assay was performed to determine cell viability. RAW264.7 macrophages were incubated with different concentrations of Sal B for 24 h. The values are presented as the means ± SEMs of three independent experiments. *N* = 6, *p* < 0.01 (**) or *P* < 0.001 (***).

An MTT assay was performed to detect the viability of cells treated with different concentrations of Sal B (0, 50, 100, 150, 200, 250, 300, 350 μM). RAW264.7 macrophages were exposed to different concentrations of Sal B for 24 h, and cell viability was found to be significantly decreased compared with that of the control group when the concentration reached 250 μM (*P* < 0.05) (Fig. 2). Therefore, 100, 150, and 200 μM Sal B were selected as the dose concentrations in subsequent experiments to prevent cytotoxicity from affecting the experimental results.

### Sal B inhibits RAW264.7 macrophages polarization to the M1 type

**Figure 3:**
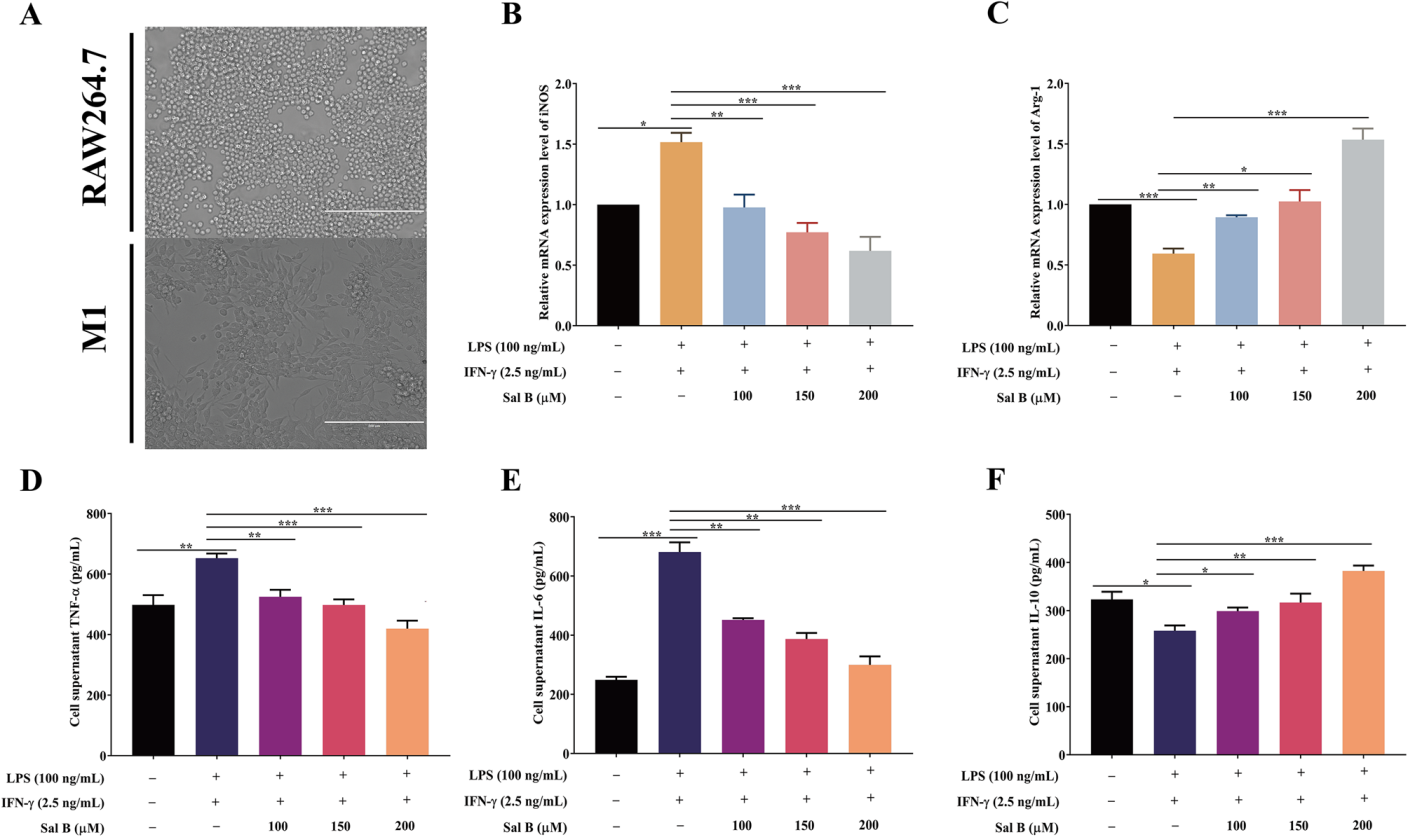
Sal B inhibits RAW264.7 macrophage M1 polarization. (**A**) Form changes after RAW264.7 macrophages were treated with LPS (100 ng/mL) + IFN-γ (2.5 ng/mL) for 24 h. RAW264.7 macrophages were treated with Sal B (100, 150 or 200 μM) for 1 h and then stimulated with LPS (100 ng/mL) + IFN-γ (2.5 ng/mL) for 24 h. (**B**,**C**) iNOS (**B**) and Arg-1 (**C**) mRNA expression was measured by RT‒qPCR. **D-F** TNF-α (**D**), IL-6 (**E**) and IL-10 (**F**) secretion expression was evaluated by ELISA. The values are presented as the means ± SEMs. *N* = 3, *p* < 0.05 (*), *p* < 0.01 (**) or *p* < 0.001 (***).

To detect the effect of Sal B on the polarization of RAW264.7 macrophages to the M1 type, we performed various verification experiments. First, LPS (100 ng/mL) + IFN-γ (2.5 ng/mL)^28^ was added to RAW264.7 macrophages for 24 h. These cells showed extended pseudopodia, and their form changed from round to oval or fusiform (Fig. 3A). In addition, compared with that in the control group, the mRNA expression of iNOS (a marker of the M1 macrophage phenotype) was increased in the M1 group (Fig. 3B). All these results indicated that the M1 macrophages had been successfully established. Subsequently, RT‒qPCR was used to examine the mRNA level changes in the expression of the M1 marker iNOS and the M2 marker Arg-1. The mRNA expression level of iNOS was significantly decreased, and that of Arg-1 was significantly increased in the Sal B treatment group compared with the M1 group (Fig. 3B,C). To further test whether Sal B inhibits RAW264.7 macrophage M1 polarization, we used ELISAs to detect TNF-α, IL-6 (M1 markers), and IL-10 (M2 markers) expression in the culture media. The ELISA results suggested that the expression levels of TNF-α and IL-6 were significantly increased and that the expression level of IL-10 was significantly decreased in the M1 group compared with the control group; however, these results were reversed when the cells were pretreated with Sal B (Fig. 3D–F).

### Sal B inhibits RAW264.7 macrophage M1 polarization by promoting autophagy

**Figure 4:**
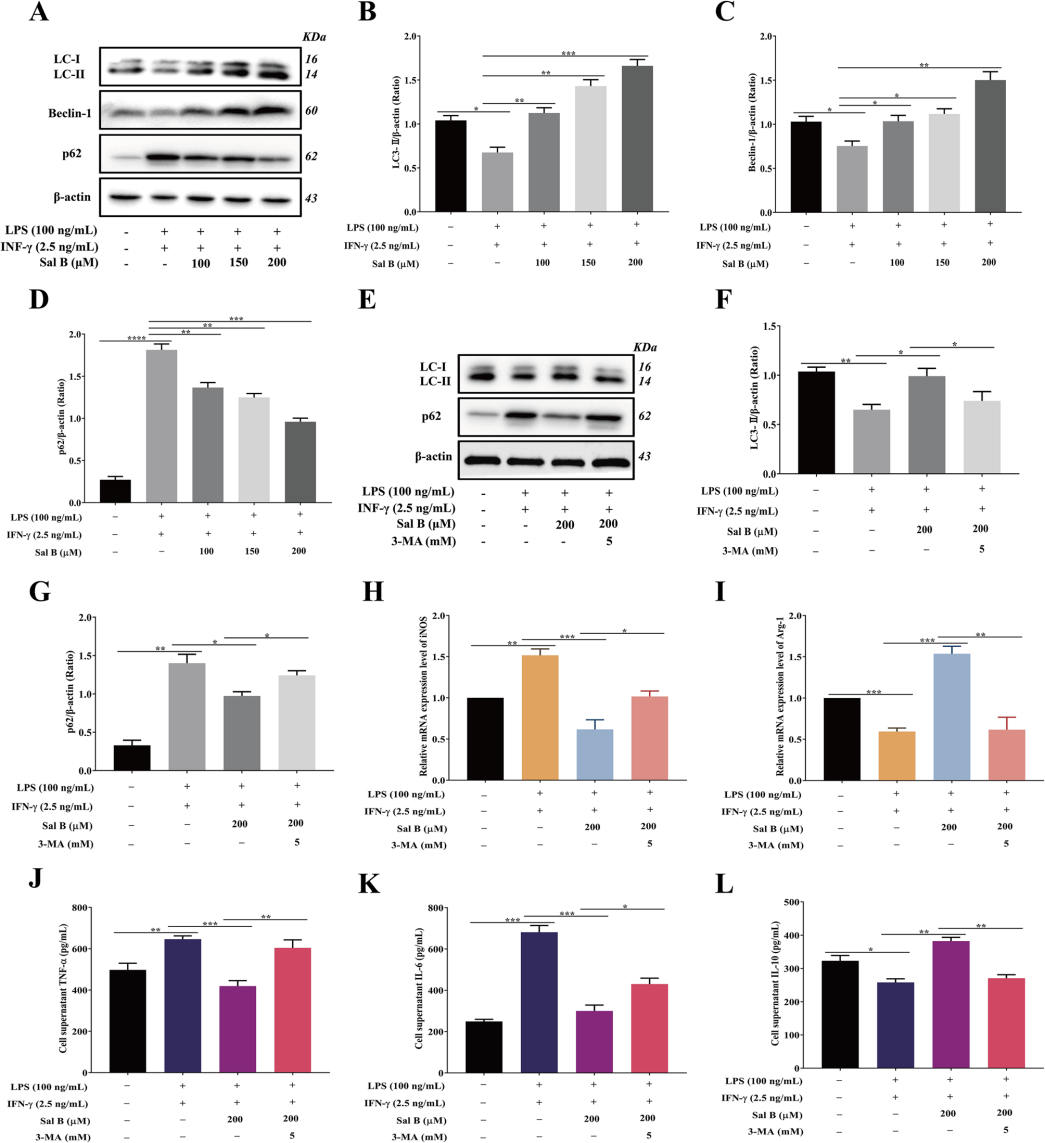
Sal B inhibits RAW264.7 macrophage M1 polarization by promoting autophagy. RAW264.7 macrophages were treated with Sal B (100, 150 and 200 μM) for 1 h and then stimulated with LPS (100 ng/mL) + IFN-γ (2.5 ng/mL) for 24 h. **A-D** LC3-II (**A**,**B**), Beclin-1 (**A**,**C**) and p62 (**A**,**D**) protein expression was analysed by western blotting. RAW264.7 macrophages were treated with Sal B (200 μM) for 1 h and then stimulated with LPS (100 ng/ml) + IFN-γ (2.5 ng/ml) + 3-MA (5 mM) for 24 h. **E–G** LC3-II (**E**,**F**) and p62 (**E**,**G**) protein expression levels were analysed by western blotting. **H**, **I** iNOS (**H**) and Arg-1 (**I**) mRNA expression levels were measured by RT‒qPCR. **J-L** TNF-α (**J**), IL-6 (**K**) and IL-10 (**L**) secretion was evaluated by ELISA. The values are presented as the means ± SEMs. *N* = 3, *p* < 0.05 (*), *p* < 0.01 (**), *p* < 0.001 (***) or *p* < 0.0001 (****).

The expression of LC3-II, Beclin-1, and p62, which are autophagy marker proteins, was measured by western blotting to determine whether Sal B promoted autophagy in M1 macrophages. The RAW264.7 macrophages were pretreated with different concentrations of Sal B (100, 150, and 200 μM) for 1 h and then incubated with LPS (100 ng/mL) + IFN-γ (2.5 ng/mL) for 24 h. The results showed that the expression levels of LC3-II and Beclin-1 were decreased and the expression level of p62 was increased in the M1 group compared with respective levels in the control group. However, compared with the M1 group, LC3-II and Beclin-1 were increased, and p62 was decreased in the Sal B treatment group (Fig. 4A–D). These changes indicated that Sal B enhances autophagy in M1 macrophages.

3-MA is a autophagy inhibitor that blocks the formation of autophagosomes by inhibiting type 1 PI3K^29^, thus inhibiting autophagy activation. To determine whether Sal B can inhibit the polarization of RAW264.7 macrophages to the M1 phenotype by activating autophagy, 3-MA was added to stimulate cells. Compared with the levels in the Sal B group, in the 3-MA treatment group, the protein expression level of LC3-II was decreased, and that of p62 was increased (Fig. 4E–G); the mRNA expression level of iNOS was significantly increased, and that of Arg-1 was significantly decreased (Fig. 4H,I); and the expression levels of TNF-α and IL-6 were increased, and the expression level of IL-10 was decreased (Fig. 4J–L).

### Sal B inhibits RAW264.7 macrophage M1 polarization by inhibiting NF-κB signalling pathway activation

**Figure 5:**
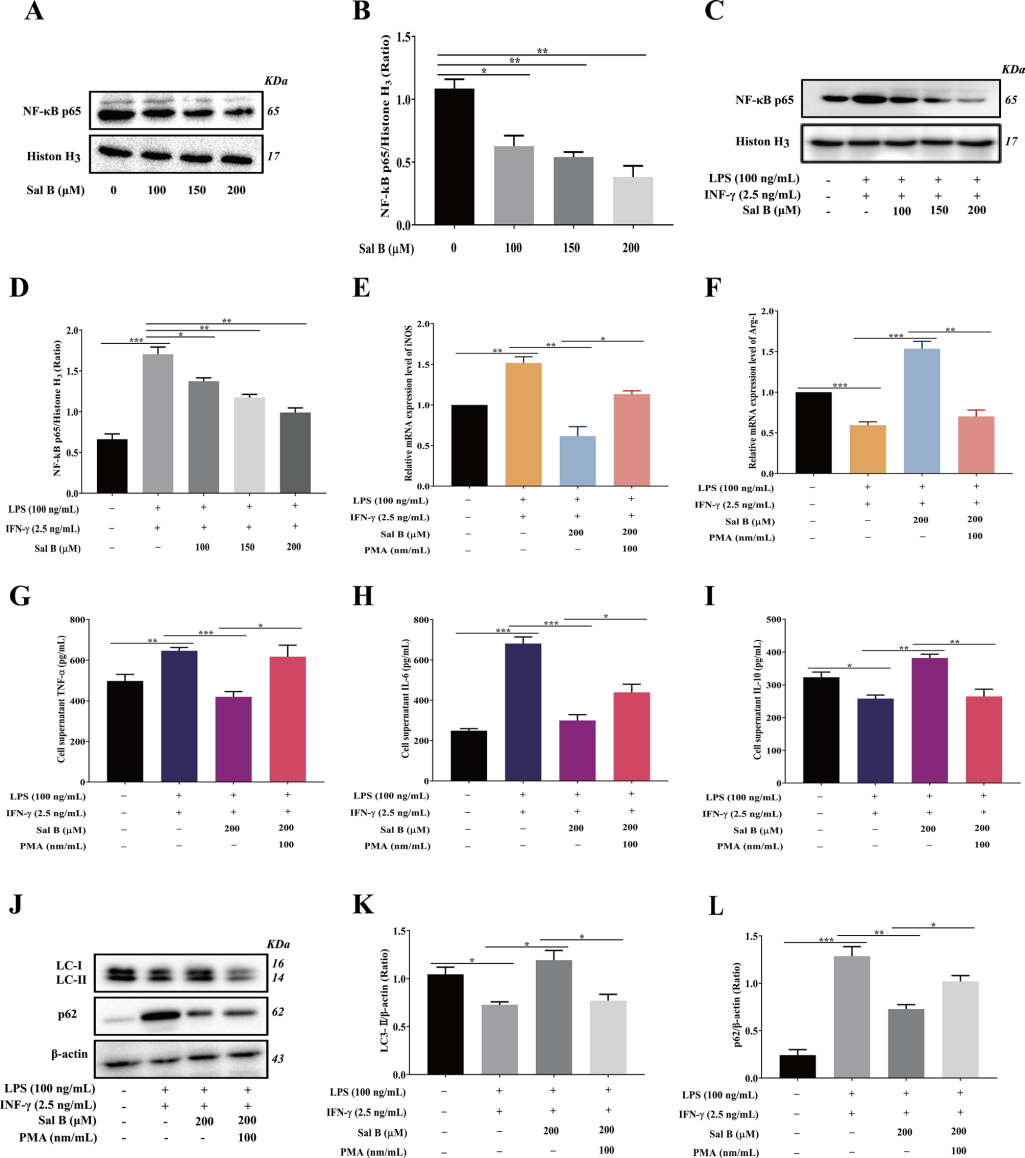
Sal B inhibits the polarization of RAW264.7 macrophages to the M1 type by inhibiting NF-κB signalling pathway activation. RAW264.7 macrophages were treated with Sal B (100, 150 or 200 μM) for 24 h, and (**A**,**B**) NF-κB p65 protein expression was analysed by western blotting. RAW264.7 macrophages were treated with Sal B (100, 150 or 200 μM) for 1 h and then stimulated with LPS (100 ng/mL) + IFN-γ (2.5 ng/mL) for 24 h, and (**C**,**D**) NF-κB p65 protein expression was analysed by western blotting. Sal B inhibits the polarization of RAW264.7 macrophages to the M1 type by inhibiting the NF-κB signalling pathway to promote autophagy. RAW264.7 macrophages were treated with Sal B (200 μM) for 1 h and then stimulated with LPS (100 ng/mL) + IFN-γ (2.5 ng/mL) + PMA (100 nm/mL) for 24 h. (**E**,**F**) iNOS (**e**) and Arg-1 (**f**) mRNA expression was measured by RT‒qPCR. **G-I** TNF-α (**G**), IL-6 (**H**) and IL-10 (**i**) secretion was evaluated by ELISA. **J-L** LC3-II (**J**,**K**) and p62 (**J**,**L**) protein expression was analysed by western blotting. The values are presented as the means ± SEMs. *N* = 3, *p* < 0.05 (*), *p* < 0.01 (**) or *p* < 0.001 (***).

To examine the influence of Sal B on the NF-κB signalling pathway, western blotting was performed to measure the protein expression of the NF-κB subunit NF-κB p65 in the nucleus. The results indicated that the protein expression of NF-κB p65 was significantly decreased in RAW264.7 macrophages treated with different concentrations of Sal B (100, 150, or 200 μM) for 24 h compared with the control group (Fig. 5A,B). After treatment with different concentrations of Sal B, the NF-κB p65 expression level was significantly decreased in the Sal B group compared with that in the M1 group (Fig. 5C,D). RT‒qPCR and ELISA were performed to examine the expression of cell markers in macrophages to determine whether Sal B inhibits RAW264.7 macrophage M1 polarization by inhibiting the NF-κB signalling pathway. The RT‒qPCR results indicated that the mRNA expression of iNOS was increased, and that of Arg-1 was decreased in the PMA group compared with the levels in the Sal B group (Fig. 5E,F). Moreover, the ELISA results indicated that the expression of TNF-α and IL-6 was increased, and the expression of IL-10 was decreased in the PMA group compared with the Sal B group (Fig. 5G–I).

### NF-κB signalling pathway activation and autophagy cooperated after Sal B treatment to inhibit RAW264.7 M1 macrophage polarization

To detect the relationship between the NF-κB pathway and autophagy in the process of Sal B treatment, western blotting was performed to measure the expression of autophagy-related proteins in macrophages pretreated with PMA (100 nm/mL) + Sal B (200 μM). The results indicated that the expression of LC3-II was decreased and that of p62 was increased in the PMA group compared with the levels in the Sal B group (Fig. 5J–L). These results indicated that activating the NF-κB pathway inhibited the activation of autophagy.

### Sal B Inhibits the Akt/mTOR pathway activated by LPS + IFN-γ

**Figure 6:**
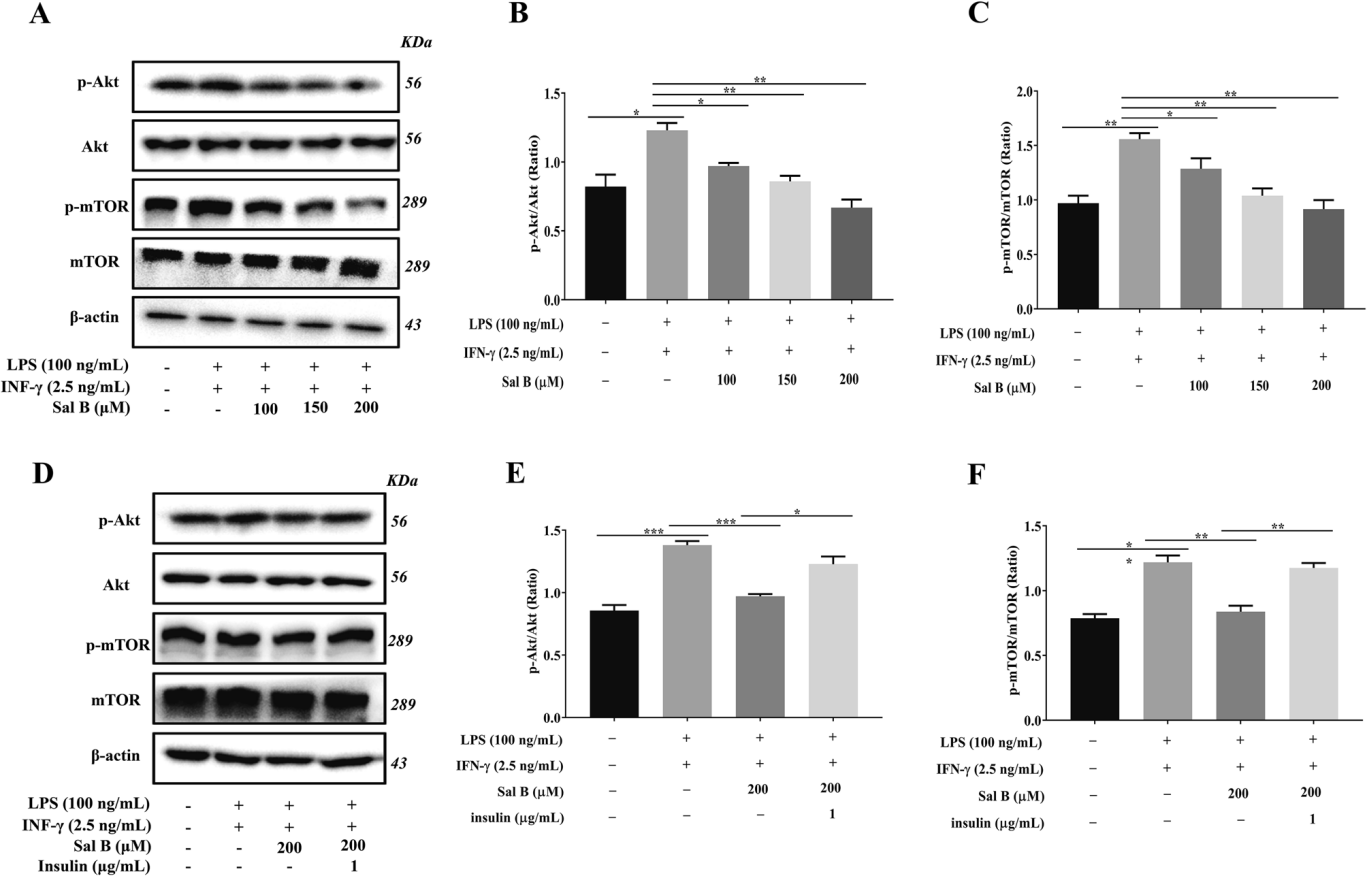
Sal B inhibits Akt/mTOR pathway activation induced by LPS + IFN-γ. RAW264.7 macrophages were treated with Sal B (100, 150 or 200 μM) for 1 h and then stimulated with LPS (100 ng/mL) + IFN-γ (2.5 ng/mL) for 24 h. **A–C** p-Akt (**A**, **B**) and p-mTOR (**A**, **C**) protein expression was analysed by western blotting. RAW264.7 macrophages were treated with Sal B (200 μM) for 1 h and then stimulated with LPS (100 ng/mL) + IFN-γ (2.5 ng/mL) + insulin (1 μg/mL) for 24 h. **D-F** p-Akt (**D**,**E**) and p-mTOR (**D**,**F**) protein expression was analysed by western blotting. The values are presented as the means ± SEMs. *N* = 3, *p* < 0.05 (*), *p* < 0.01 (**) or *p* < 0.001 (***).

Akt/mTOR is an important signalling pathway that controls autophagy activation. Downregulating the phosphorylation of Akt inhibited mTOR signalling pathway phosphorylation, which inhibited autophagy activation. To examine the effect of Sal B on the autophagy Akt/mTOR signalling pathway in M1 macrophages, Western blotting was performed to measure the phosphorylation levels of Akt and mTOR. The results indicated that the expression levels of p-Akt and p-mTOR were increased in the M1 group compared with the control group. However, in the group pretreated with Sal B, the expression of p-Akt and p-mTOR was decreased compared with that in the M1 group (Fig. 6A–C). Moreover, to discuss the effect of Sal B on the phosphorylation levels of Akt and mTOR in M1 macrophages, M1 macrophages were pretreated with Sal B (200 μM) for 1 h, followed by LPS (100 ng/mL) + IFN-γ (2.5 ng/mL) + insulin (1 μg/mL, a AKT/mTOR signalling pathway agonist) for 24 h. The results indicated that the expression of p-AKT and p-mTOR was increased in the insulin group compared with the Sal B group (Fig. 6D–F).

### Sal B inhibits RAW264.7 macrophages M1 polarization by mediating Akt/mTOR pathway activation

**Figure 7:**
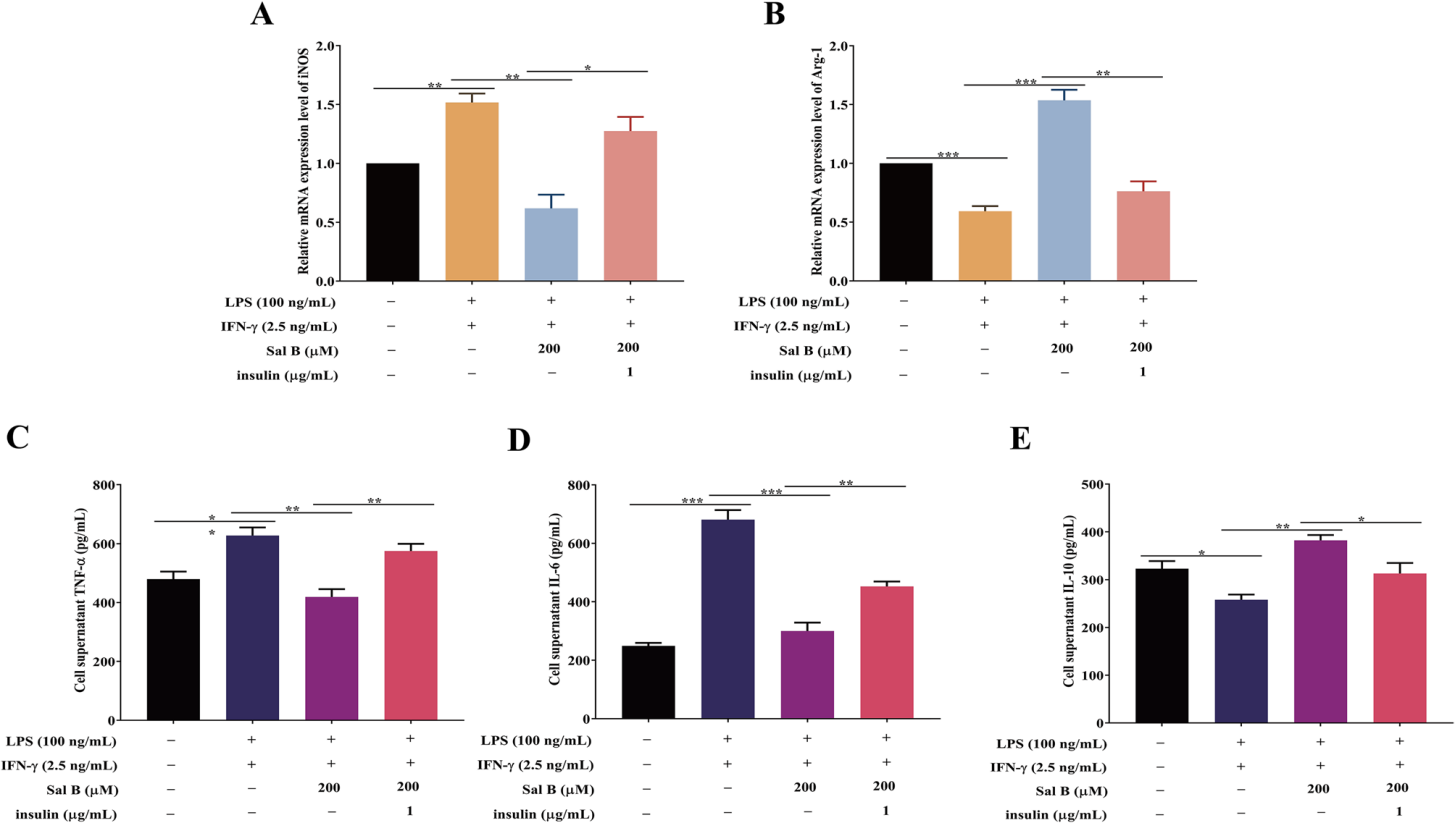
Sal B inhibits RAW264.7 macrophage M1 polarization through the Akt/mTOR pathway. RAW264.7 macrophages were treated with Sal B (200 μM) for 1 h and then stimulated with LPS (100 ng/mL) + IFN-γ (2.5 ng/mL) + insulin (1 μg/mL) for 24 h. **A**,**B** iNOS (**A**) and Arg-1 (**B**) mRNA expression was measured by RT‒qPCR. **C-E** TNF-α (**C**), IL-6 (**D**) and IL-10 (**E**) secretion expression was evaluated by ELISA. The values are presented as the means ± SEMs. *N* = 3, *p* < 0.05 (*), *p* < 0.01 (**) or *p* < 0.001 (***).

**Figure 8:**
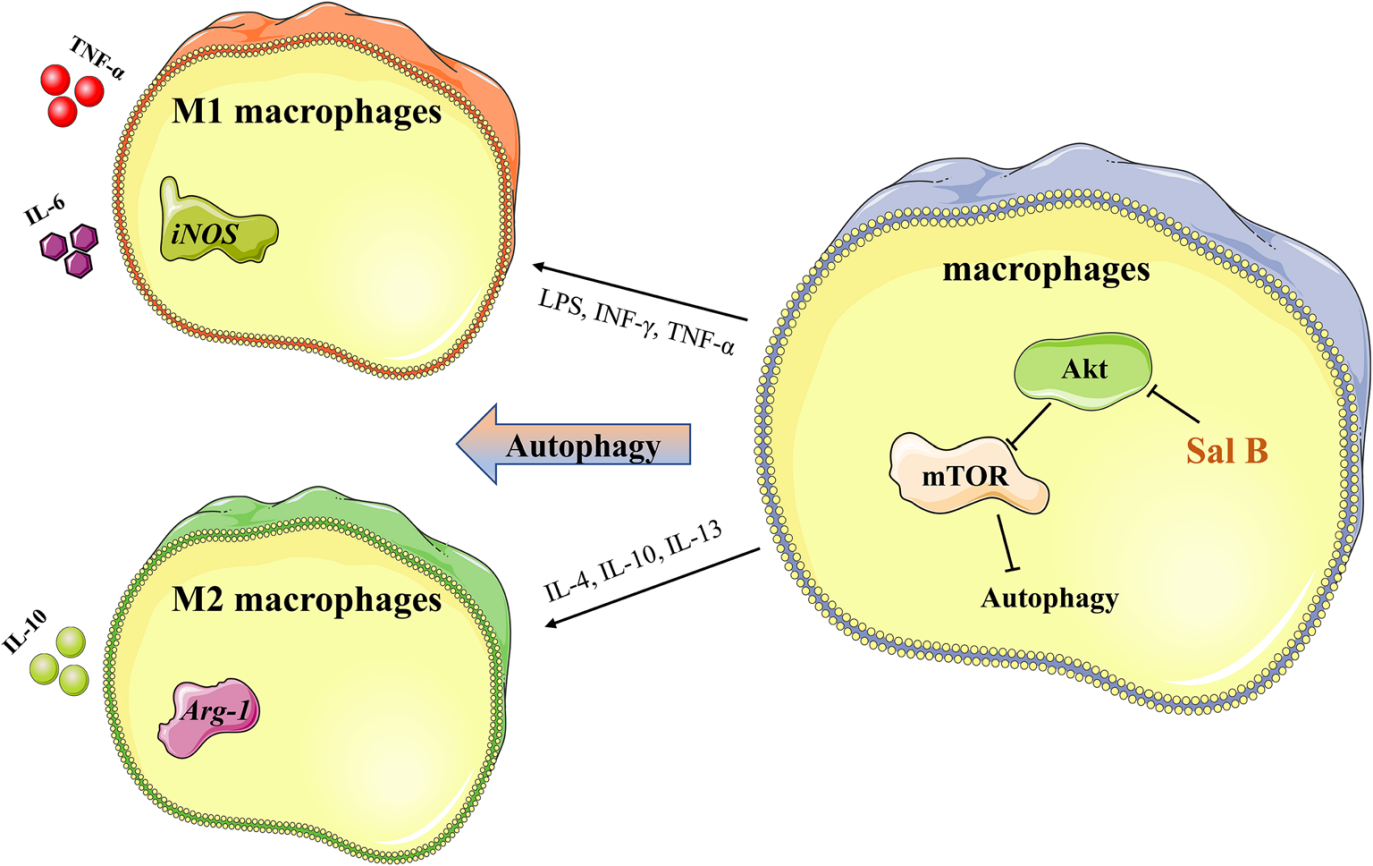
A schematic diagram of the mechanism of Sal B on macrophages.

To investigate whether the activation of the autophagic Akt/mTOR signalling pathway can completely reverse the inhibition of Sal B in macrophage M1 polarization, RT‒qPCR was performed, and the results indicated that the mRNA expression of iNOS was increased and that of Arg-1 was decreased in the insulin group compared with the Sal B group (Fig. 7A,B). The ELISA results indicated that in the insulin group, the expression of TNF-α and IL was increased and that of IL-10 was decreased compared with levels in the Sal B group (Fig. 7C–E). These results suggest that insulin activation of the Akt/mTOR pathway partially offset the inhibitory effect of Sal B on M1 macrophage polarization, indicating that Sal B inhibits M1 macrophage polarization by partially inhibiting Akt/mTOR pathway activation. A schematic diagram of the mechanism of Sal B on macrophages (Fig. 8).

## Discussion

AS serves is a pathological basis of cardiovascular diseases, and the incidence of AS has been increasing every year, seriously endangering human life and health^30^. Several studies have shown that macrophages play important roles in the development of AS^31–33^. It has been shown that the macrophage-mediated inflammatory response is one of the mechanisms in the occurrence and development of AS, in which the macrophage phenotype plays a vital role^34^. IFN-γ, TNF-α, and LPS can induce the formation of M1 macrophages, which produce many proinflammatory factors, such as TNF-α and IL-6^35^, leading to the progression and enlargement of atherosclerotic plaques^36,37^. M2 macrophages are activated by IL-4, IL-13, glucocorticoids, and other stimuli, and they secrete anti-inflammatory factors such as IL-10 and TGF-β^38^ that exert an antiatherogenic effect via inflammation resolution, efferocytosis and tissue repair^39,40^. Therefore, macrophage phenotype studies are of great significance for the prevention and treatment of AS, particularly the induction of anti-inflammatory effects.

Many studies have confirmed that Sal B shows anti-inflammatory, antioxidative, and suppression of apoptosis action; therefore, it is presumed to exert an anti-atherosclerotic effect^41–45^. Lee et al^46^ found that Sal B inhibited the proliferation and migration of vascular cells by activating NF-E2-related factor 2, thus playing an anti-atherosclerotic effect. Regarding the anti-inflammatory effects of Sal B, a study^47^ has shown that Sal B inhibits the production and release of inflammatory factors TNF-α, IL-6, IL-8, etc. However, whether Sal B can prevent and treat AS by regulating the polarization state and function of macrophages and the molecular mechanism of this possible action have not been reported. To explore whether Sal B can regulate the polarization macrophages, we designed a series of experiments. To establish model cell types, LPS (100 ng/mL) and IFN-γ (2.5 ng/mL were used to pretreat RAW264.7 macrophages for 12 h as reported in the literature^28^, and the expression of the M1 macrophage markers iNOS, TNF-α and IL-6 were found to be significantly increased compared with that in the control group. Moreover, the cell morphology was observed under the microscope, and significant changes were evident, suggesting that the model concentration was appropriate for subsequent experiments. The MTT results showed that Sal B treated at concentrations less than 200 μM exerted little impact on cell viability; therefore, 200 μM, 150 μM and 100 μM were selected as the dose concentrations. Subsequently, RT‒qPCR and ELISA were performed to evaluate whether Sal B inhibits M1 macrophage polarization. The results indicated that pretreatment with Sal B decreased the production and release of M1 macrophage markers (iNOS, TNF-α and IL-6) and increased the expression of M2 markers (Arg-1 and IL-10).Autophagy, a programmed process of cell degradation, not only removes damaged organelles or toxic proteins but also provides essential materials and energy for the body and cells^48^. A recent study suggests that macrophage autophagy enhancement exerts a protective effect in AS^49,50^. In the regulation of macrophage polarization by Sal B, a study demonstrated that autophagy played an important role^51^. Despite evidence showing that macrophage autophagy deficiency led to increased atherosclerosis, the molecular mechanism by which Sal B inhibited RAW264.7 macrophage M1 polarization was unknown. Therefore, western blotting was performed to examine the expression of the autophagy-related proteins LC3-II and p62 to explain the molecular mechanism of Sal B inhibition of RAW264.7 macrophage M1 polarization. In the present study, compared with the control group, in the M1 group, LC3-II expression decreased significantly and led to a significant increase in p62 expression, suggesting that autophagy was inhibited during macrophage M1 polarization, which was consistent with the study of Li et al.^14^. However, after treatment with Sal B for 24 h, the expression of LC3-II increased and that of p62 decreased significantly compared with that in the M1 group, which showed that Sal B enhanced autophagy during macrophage M1 polarization. Additionally, compared with that in the Sal B group, the protein abundance of LC3-II decreased and that of p62 increased significantly, and the abundance of e iNOS, TNF-α and IL-6 was increased, and that was Arg-1 and IL-10 was decreased in the 3-MA group. These results demonstrated that 3-MA not only reversed the inductive effect of Sal B on macrophages but also partially reversed the inhibitory effect of Sal B on macrophage M1 polarization.

The NF-κB pathway, a classical pathway that regulates inflammation in the body, has been extensively studied in the inflammatory process. Wang et al.^52^ demonstrated that tanshinone I selectively inhibited the production of LPS-induced proinflammatory factors such as TNF-α and IL-6 by inhibiting NF-κB in mice with Parkinson's disease. Li et al.^53^ demonstrated that paeoniflorin inhibited the secretion and production of inflammatory factors in the process of VSMC proliferation and migration by inhibiting NF-κB expression. Wang HW et al.^54^ showed that salidroside decreased the expression of iNOS, COX_2_, IL-1β, IL-6, and TNF-α by inhibiting NF-κB pathway activation. In addition, Wang et al.^55^ demonstrated that Sal B alleviated angiotensin II and induced cardiac fibrosis by suppressing NF-κB pathway activation, playing a protective role in the myocardium. These studies suggested that the NF-κB signalling pathway may have played an important role in Sal B inhibition of macrophages M1 polarization. To investigate the role played by the NF-κB signalling pathway in this process, first, RAW264.7 macrophages treated with Sal B showed decreased NF-κB p65 expression, and the same reduction in NF-κB p65 protein level was found after we added LPS (100 ng/mL) + IFN-γ (2.5 ng/mL) to the cultures. These findings indicated that the NF-κB signalling pathway was inhibited during polarization. Then, the mRNA expression of iNOS was increased and that of Arg-1 was decreased in the PMA group compared with the Sal B group. PMA has been shown to induce canonical NF-κB-dependent transcription by acting through the direct activation of protein-kinase-C^56,57^. Moreover, the ELISA results indicated that the TNF-α and IL-6 levels were increased, and the IL-10 level was decreased. Xia et al.^58^ observed the effect of Sal B on the expression of inflammatory cytokines in rheumatoid arthritis and found that Sal B inhibited the production and release of inflammatory cytokines through the NF-κB signalling pathway. Studying atherosclerosis, Xu et al.^59^ confirmed that Sal B inhibited the production and release of proinflammatory factors by inhibiting the activation of the NF-κB signalling pathway in vitro. The conclusions of these studies confirm the conclusions of our experiment. Regarding the relationship between the NF-κB signalling pathway and autophagy, the expression of LC3-II decreased and that of p62 increased significantly when PMA was added, suggesting that activation of the NF-κB pathway inhibited autophagy.

The signal transduction pathway of phosphatidylinositol 3 kinase/protein kinase B/mammalian target of rapamycin (PI3K/Akt/mTOR) is an important signalling pathway in autophagy that can affect the initiation and termination of autophagy^60^. Bai et al.^61^ showed that LZ205 exerted an anti-inflammatory effect by inhibiting M1 macrophage polarization by regulating the PI3K/Akt/mTOR signalling pathway. We designed an experiment to explore whether autophagy is enhanced through the Akt/mTOR signalling pathway during the inhibition of macrophage M1 polarization induced by Sal B. In the present study, treatment with LPS (100 ng/mL) + IFN-γ (2.5 ng/mL) significantly increased the levels of p-Akt and p-mTOR, but pretreatment with Sal B significantly attenuated the increases in p-Akt and p-mTOR. This finding indicated that M1 polarization activated the Akt/mTOR pathway and that Sal B suppressed autophagy enhancement during M1 macrophage polarization by inhibiting Akt/mTOR pathway activation. In addition, the expression levels of p-Akt and p-mTOR were significantly increased in the insulin group compared with the Sal B group. Simultaneously, the iNOS, TNF-α, and IL-6 levels were increased, and the Arg-1 and IL-10 levels were decreased. The downregulating effect of Sal B on Akt/mTOR pathway activation was inhibited after treatment with insulin (an activator of the Akt/mTOR pathway), and the effect of Sal B on the inhibition of macrophage M1 polarization was also partially abrogated. Therefore, Sal B partially inhibits macrophage M1 polarization by downregulating Akt/mTOR pathway activation.

## Conclusions

In conclusion, autophagy is inhibited during the polarization of macrophages to the M1 phenotype, and Sal B inhibited M1 macrophages polarization and promoted M2 macrophage polarization. In this process, Sal B mainly inhibited NF-κB signalling pathway activation and downregulated Akt/mTOR signaling to promote autophagy via this molecular mechanism, and NF-κB activation inhibited autophagy. This study shows that Sal B may show high anti-atherosclerotic effects by regulating the polarization state of macrophages in vitro. In the future, we will carry out experiments to further explore and verify these findings in vivo.

## Supplementary Information

Supplementary Information 1.

Supplementary Information 2.

## Data Availability

The datasets used in the current study are included in the published article or available from the corresponding author on reasonable request.
