# Supplementary material for: Salvianolic acid B inhibits RAW264.7 cell polarization towards the M1 phenotype by inhibiting NF-κB and Akt/mTOR pathway activation
Source: Sci Rep. 2022 Aug 16;12:13857. doi: 10.1038/s41598-022-18246-0 (PMC9381594; doi:10.1038/s41598-022-18246-0)
Supplement: Supplementary file 1 — Supplementary Information 1. [file 41598_2022_18246_MOESM1_ESM.docx]

**Experimental design**

Sal B was dissolved in DMSO at 1 mM, stored in aliquots at -20 °C, and further diluted (as appropriate)

|  | LPS | IFN-γ | Sal B | 3-MA | PMA | Insulin |
| --- | --- | --- | --- | --- | --- | --- |
| Control | - | - | - | - | - | - |
| M1 group | + | + | - | - | - | - |
| Sal B group | + | + | + | - | - | - |
| 3-MA group | + | + | + | + | - | - |
| PMA group | + | + | + | - | + | - |
| Insulin group | + | + | + | - | - | + |

in culture medium. The following cell groups were generated:
