# Supplementary material for: Salvianolic acid B inhibits RAW264.7 cell polarization towards the M1 phenotype by inhibiting NF-κB and Akt/mTOR pathway activation
Source: Sci Rep. 2022 Aug 16;12:13857. doi: 10.1038/s41598-022-18246-0 (PMC9381594; doi:10.1038/s41598-022-18246-0)

Original uncropped images of western blots used for the experiment.

Figure 4A

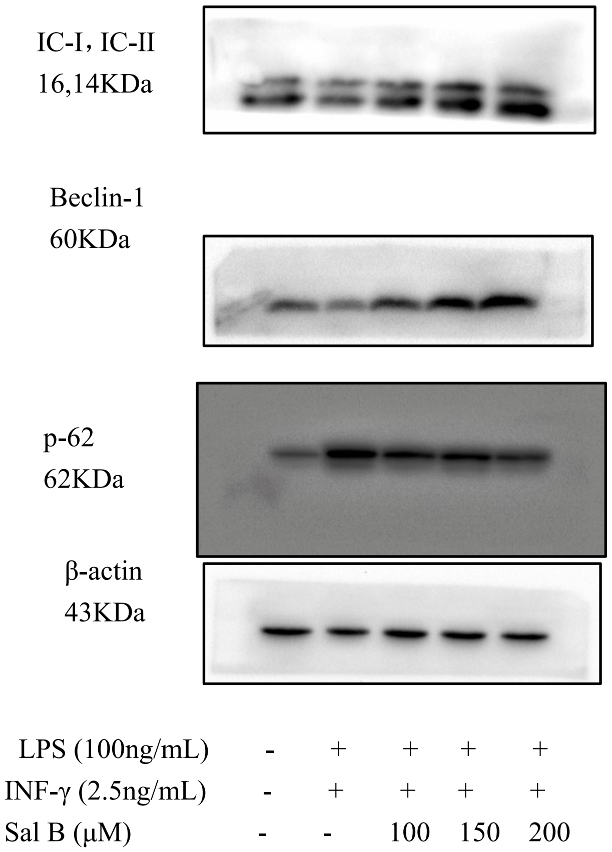

Figure 4E

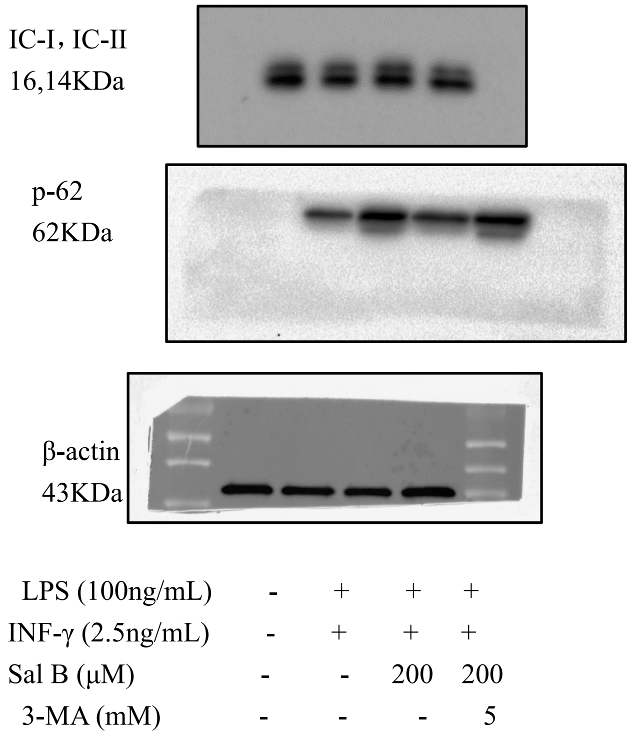

Figure 5A

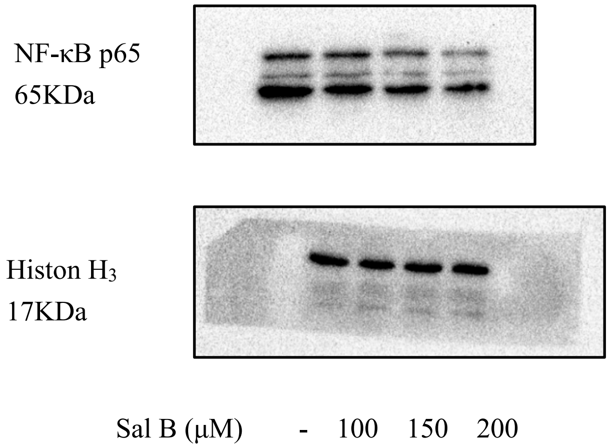

Figure 5C

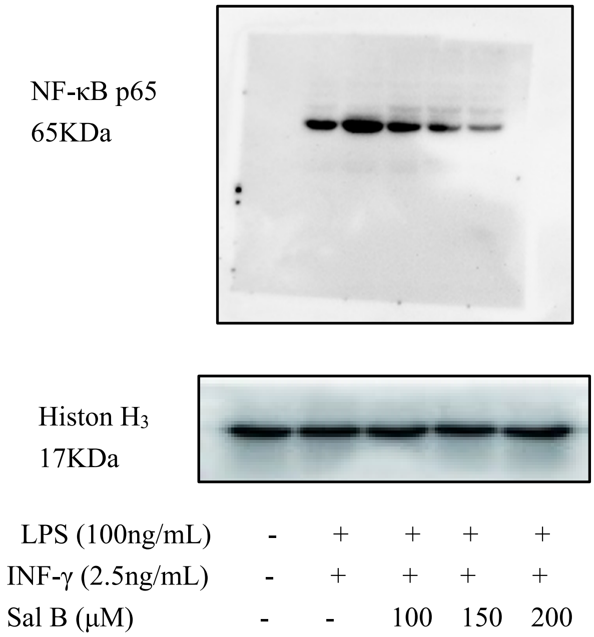

Figure 5J

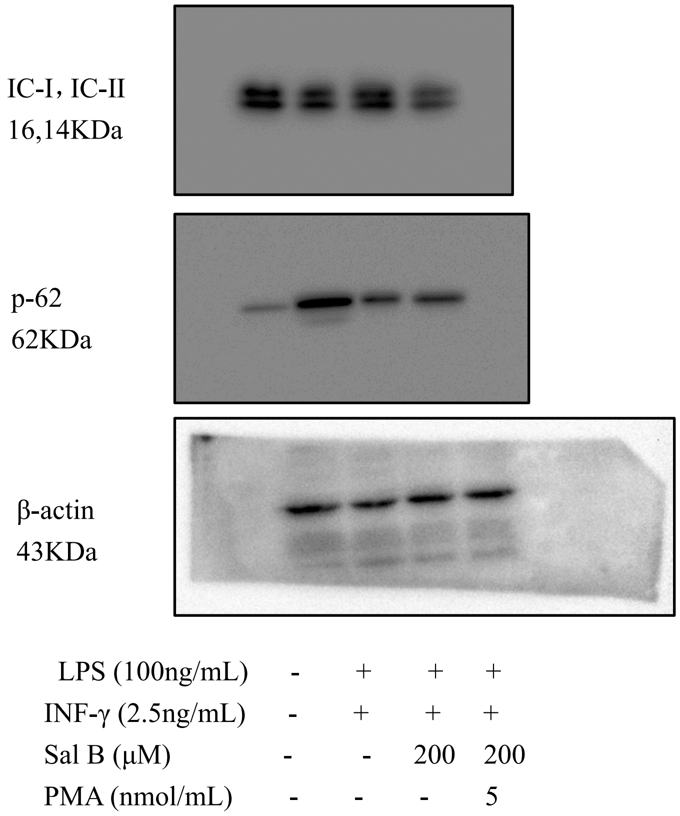
IC-I, IC-II
16,14KDa
p-62
62KDa
β-actin
43KDa
LPS (100ng/mL) - + + +
INF-γ (2.5ng/mL) - + + +
Sal B (μM) - - 200 200
PMA (nmol/mL) - - - 5

Figure 6A

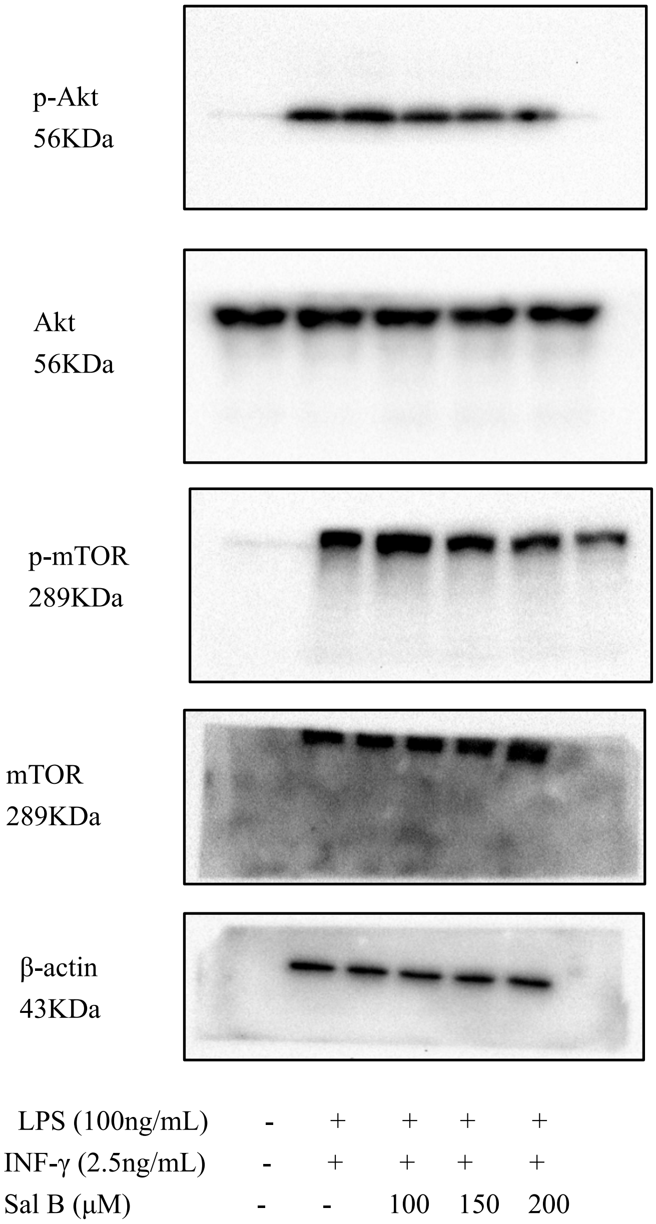

Figure 6D

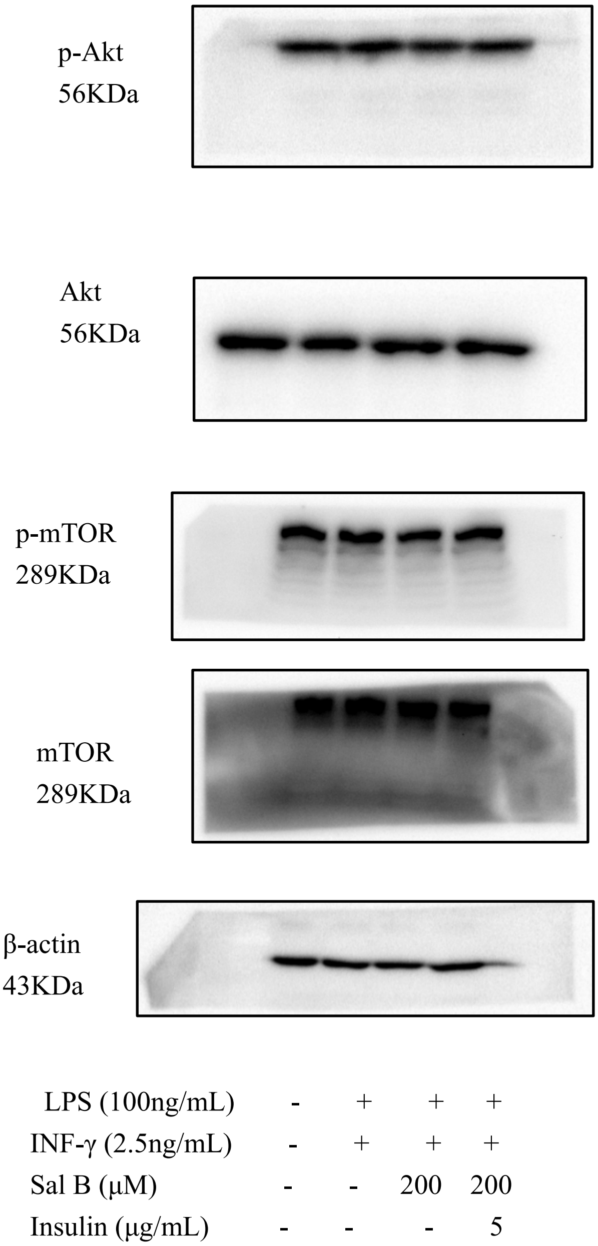

Supplement: Supplementary file 2 — Supplementary Information 2. [file 41598_2022_18246_MOESM2_ESM.pdf]
